# TNFR1-JNK signaling is the shared pathway of neuroinflammation and neurovascular damage after LPS-sensitized hypoxic-ischemic injury in the immature brain

**DOI:** 10.1186/s12974-014-0215-2

**Published:** 2014-12-24

**Authors:** Lan-Wan Wang, Ying-Chao Chang, Shyi-Jou Chen, Chien-Hang Tseng, Yi-Fang Tu, Nan-Shih Liao, Chao-Ching Huang, Chien-Jung Ho

**Affiliations:** Department of Pediatrics, Chi Mei Medical Center, Tainan, 710 Taiwan; Department of Pediatrics, College of Medicine, Taipei Medical University, #250, Wu-Hsing Street, Taipei, 11031 Taiwan; Department of Pediatrics, Wan-Fang Hospital, Taipei Medical University, Taipei, 110 Taiwan; Department of Pediatrics, School of Medicine, Chung Shan Medical University, Taichung, 402 Taiwan; Department of Pediatrics, Chang Gung Memorial Hospital - Kaohsiung Medical Center, Chang Gung University College of Medicine, Kaohsiung, 833 Taiwan; Department of Pediatrics, Tri-Service General Hospital, National Defense Medical Center, Taipei, 114 Taiwan; Department of Pediatrics, College of Medicine, National Cheng Kung University Hospital, National Cheng Kung University, Tainan, 704 Taiwan; Institute of Molecular Biology, Academia Sinica, Taipei, 115 Taiwan

**Keywords:** Tumor necrosis factor receptor 1, c-Jun N-terminal kinase, Neurovascular unit, Neuroinflammation, Lipopolysaccharide, Hypoxic-ischemia, Immature brain

## Abstract

**Background:**

Hypoxic-ischemia (HI) and inflammation are the two major pathogenic mechanisms of brain injury in very preterm infants. The neurovascular unit is the major target of HI injury in the immature brain. Systemic inflammation may worsen HI by up-regulating neuroinflammation and disrupting the blood–brain barrier (BBB). Since neurons and oligodendrocytes, microvascular endothelial cells, and microglia may closely interact with each other, there may be a common signaling pathway leading to neuroinflammation and neurovascular damage after injury in the immature brain. TNF-α is a key pro-inflammatory cytokine that acts through the TNF receptor (TNFR), and c-Jun N-terminal kinases (JNK) are important stress-responsive kinases.

**Objective:**

To determine if TNFR1-JNK signaling is a shared pathway underlying neuroinflammation and neurovascular injury after lipopolysaccharide (LPS)-sensitized HI in the immature brain.

**Methods:**

Postpartum (P) day-5 mice received LPS or normal saline (NS) injection before HI. Immunohistochemistry, immunoblotting and TNFR1- and TNFR2-knockout mouse pups were used to determine neuroinflammation, BBB damage, TNF-α expression, JNK activation, and cell apoptosis. The cellular distribution of p-JNK, TNFR1/TNFR2 and cleaved caspase-3 were examined using immunofluorescent staining.

**Results:**

The LPS + HI group had significantly greater up-regulation of activated microglia, TNF-α and TNFR1 expression, and increases of BBB disruption and cleaved caspase-3 levels at 24 hours post-insult, and showed more cortical and white matter injury on P17 than the control and NS + HI groups. Cleaved caspase-3 was highly expressed in microvascular endothelial cells, neurons, and oligodendroglial precursor cells. LPS-sensitized HI also induced JNK activation and up-regulation of TNFR1 but not TNFR2 expression in the microglia, endothelial cells, neurons, and oligodendrocyte progenitors, and most of the TNFR1-positive cells co-expressed p-JNK. Etanercept (a TNF-α inhibitor) and AS601245 (a JNK inhibitor) protected against LPS-sensitized HI brain injury. The TNFR1-knockout but not TNFR2-knockout pups had significant reduction in JNK activation, attenuation of microglial activation, BBB breakdown and cleaved caspase-3 expression, and showed markedly less cortical and white matter injury than the wild-type pups after LPS-sensitized HI.

**Conclusion:**

TNFR1-JNK signaling is the shared pathway leading to neuroinflammation and neurovascular damage after LPS-sensitized HI in the immature brain.

**Electronic supplementary material:**

The online version of this article (doi:10.1186/s12974-014-0215-2) contains supplementary material, which is available to authorized users.

## Background

Human studies and animal models have provided evidence on the detrimental roles of hypoxic-ischemia (HI) and inflammation to the brain of very preterm infants [1-3]. Preterm infants experience various HI and infectious insults during the neonatal period, with such infections predisposing to or aggravating HI. Clinical studies have shown that increased levels of systemic cytokines in premature infants with chorioamnionitis are associated with hemodynamic disturbances leading to cerebral HI, whereas co-morbid chorioamnionitis and placental perfusion defects put preterm infants at higher risk of abnormal neurologic outcomes than either insult alone [4-6]. HI and infectious events across the perinatal and neonatal periods have cumulative effects on the risk of cerebral palsy in very preterm infants [7]. Animal studies also show that pre-exposure to systemic lipopolysaccharide (LPS) sensitizes HI injury in the immature brain [8,9].

The major target of ischemia-reperfusion injury is the neurovascular unit, which is composed of neurons, oligodendroglia and microvessels [9,10]. Neuronal or oligodendroglial apoptosis, microvascular damage, that is blood–brain barrier (BBB) disruption, and microglial activation have been linked to the severity of HI injury in the immature brain [8,9,11,12]. For very premature infants, the O4-positive pre-myelinating oligodendrocyte progenitors are the target cells of white matter injury [13]. Activated microglia are the hallmark of neuroinflammation and may exacerbate brain injury through damage to the neurovascular unit [14,15]. During detrimental insults, activated microglia may induce gray matter and white matter injury through the production of pro-inflammatory cytokines, such as TNF-α [1]. The damaged microvessels may recruit activated leukocytes into the injured brain through the disrupted BBB, resulting in sustained activation of microglia, which in turn cause further damage through prolonged production of pro-inflammatory cytokines [15]. Since neurons and oligodendrocyte progenitors, microvascular endothelial cells, and microglia may closely interact with each other, there may be a common signaling pathway leading to neuroinflammation and neurovascular damage after insults. Therefore, blocking such common signal transduction to reduce neuroinflammation and attenuate neurovascular damage may effectively provide neuroprotection to the immature brain.

c-Jun N-terminal kinases (JNK) are important stress-responsive kinases [16]. Our previous study has shown that JNK signaling is the shared pathway linking neuroinflammation, BBB disruption, and oligodendroglial apoptosis in the white matter injury of the immature rat brain [9]. However, the upstream pathway leading to JNK activation in neuroinflammation and in the cells of the neurovascular unit remains unclear. TNF-α is a key pro-inflammatory cytokine in the pathogenesis of brain injury in premature infants [1,17,18], TNF-α and JNK activation precede cell death by inflammation and apoptosis [16,19]. TNF-α signaling triggers inflammatory gene expression and JNK-mediated intrinsic/extrinsic apoptotic cascades, while JNK activation can further stimulate TNF-α synthesis through AP-1 transcription [16,19]. TNF-α exerts its biologic effects by signaling through two receptors, the TNF receptor (TNFR) 1 and TNFR2, which are detected in affected brain areas in preterm infants with periventricular leukomalacia [18]. Constitutively expressed in various neural and endothelial cells, TNFR1 are involved in inflammatory and apoptotic processes, while TNFR2 expression is often induced by injury and may have the opposite effects [19,20]. TNF-α and JNK activation have been respectively reported to play roles in microglia-mediated neuroinflammation or BBB permeability [1,9,21]. Inhibition of either TNF-α-TNFR1 or JNK signaling exerts neuroprotective effects against HI brain injury in animal experiments [22,23]. Therefore, TNF-α and JNK signal transduction may have cross-talk in the pathogenesis of HI-induced neuroinflammation and neurovascular injury in the immature brain.

Our studies have demonstrated that JNK activation plays important roles in LPS-sensitized HI injury in the neurovascular unit of the immature rat brain [8,9]. However, it remains unclear whether TNFR1-JNK signaling is the shared pathway linking neuroinflammation and neurovascular damage after LPS-sensitized HI. Thus, using pharmacologic and genetic approaches in postpartum (P) day-5 mouse pups (brain maturation status equivalent to human gestation < 30 weeks), this study tested the hypothesis that TNFR1-JNK signaling is a shared pathway leading to neuroinflammation, microvascular damage, and neuronal and oligodendrocyte progenitor apoptosis in LPS-sensitized HI injury of the immature brain.

## Methods

### Establishing a mouse model of LPS-sensitized hypoxic-ischemic injury in the immature brain

This animal study was approved by the Animal Care Committee of National Cheng Kung University. The experimental paradigm adapted from Vannucci’s HI method [24] was modified to include LPS sensitization in P5 mice. The brain maturation of P5 mice developmentally corresponds to very preterm infants, and has apoptotic and cell death mechanisms characteristic of the immature brain after HI [25]. P5 mouse pups (C57BL/6) were first injected intraperitoneally (ip) with LPS (*Escherichia coli* 055:B5; Sigma-Aldrich, St Louis, MO, USA) or pyrogen-free normal saline (NS). The pups were then randomly assigned to 3 different groups: control (NS injected without HI), NS + HI (NS injected 3 hours before HI), and LPS + HI (LPS 0.05 mg/kg injected 3 hours before HI). To avoid LPS-induced body temperature changes, the mouse pups were returned to their dams after LPS or NS injection, and housed in an incubator to maintain body temperature at 33 to 34°C before HI.

The HI was induced by right carotid artery ligation followed by hypoxia [8,9]. The right common carotid artery was permanently ligated under 2.5% halothane anesthesia. The average length of surgery to occlude the artery was 2 minutes. After surgery, the pups were put into an incubator for a 1-hour recovery. They were then placed in airtight 500-mL containers partially submerged in a 36°C water bath, with humidified 8% oxygen kept at a flow rate of 3 L/minute for 30 minutes. Following hypoxia, the pups were returned to their dam. Technicians performed the experiments, while investigators blinded to the grouping performed the quantitative measurements.

### Pharmacological inhibition of TNF-α

Etanercept is a non-selective TNF-α inhibitor that prevents TNF-α binding to TNFR by neutralizing the actions of soluble and transmembrane TNF-α [26]. The P5 mouse pups were randomly assigned to the control group (without exposure to LPS + HI), and the 3 LPS + HI groups that received ip injection of 5 or 15 mg/kg of etanercept (Enbrel, Wyeth Europa Ltd., Maidenhead, Berkshire, UK) or vehicle (NS) at 30 minutes before, immediately, and 3 hours after LPS + HI. The etanercept doses used were modified from Aden’s study [27].

### TNFR1/TNFR2 knockout mice

TNFR1- and TNFR2-knockout (KO) mice were bought from the Jackson Laboratory (Bar Harbor, ME, USA). The donor strains of TNFR1- and TNFR2-KO mice were from 129S2 via D3 ES cell line with a homozygous × homozygous mating system with a C57BL/6 genetic background.

### Pharmacological inhibition of JNK

AS601245, a highly specific JNK inhibitor, blocks JNK activity by binding to its ATP-binding site [28]. The P5 pups were randomly assigned to the control group (without exposure to LPS + HI) and the 3 LPS + HI groups that received ip injection of 20 or 40 mg/kg of AS601245 (Alexis Biochemicals, Lausen, Switzerland) or vehicle (dimethyl sulfoxide (DMSO), Sigma-Aldrich, St Louis, MO, USA) at 30 minutes before and immediately after LPS + HI. The doses of AS601245 used were modified from Carboni’s study [28].

### Western blot analysis

The ipsilateral hemisphere was homogenized in cold lysis buffer and the protein concentrations were determined using a Bio-Rad Protein Assay kit (Bio-Rad Laboratories, Hercules, CA, USA). Samples (50 μg) were separated using 10% SDS-PAGE and blotted onto polyvinylidene fluoride membranes. The membranes were incubated with primary antibodies. Immunoreactivity was detected by horseradish-conjugated secondary antibodies and visualized by enhanced chemiluminescence. The primary antibodies used were anti-TNF-α (1:500; Biolegend, San Diego, CA, USA), anti-phospho-JNK (p-JNK) (Thr183/Tyr185, 1:1,000; Cell Signaling, Danvers, MA, USA), anti-cleaved caspase 3 (1:1,000; Cell Signaling, Danvers, MA, USA), and anti-β-actin (1:5,000; Invitrogen, Carlsbad, CA, USA). The band signals were quantified using an imaging software (ImagePro Plus 6.0; Media Cybernetics, Bethesda, MD, USA).

### Immunohistochemistry

Mouse pups were sacrificed and perfused for cryosections on P6 (24 hours post-insult). The brains were post-fixed, dehydrated using 30% (w/v) sucrose in PBS, and coronally sectioned (20-μm thick) from the genu of the corpus callosum to the end of the dorsal hippocampus. Three sections per brain, one at the level of the striatum (0.14 mm anterior to the bregma) and another 2 at the levels of the dorsal hippocampus (1.94 mm and 2.54 mm posterior to the bregma) according to a mouse brain atlas [29], were selected for immunohistochemical staining.

Immunohistochemistry was performed for microglial activation (ionized calcium-binding adaptor molecule-1, Iba-1), immunoglobulin G (IgG) extravasation, cleaved caspase-3, p-JNK, TNFR1 and TNFR2. IgG extravasation was used as an indicator of BBB permeability [30]. After eradication of endogenous peroxidases and blocking of non-specific binding, brain sections were incubated at 4°C overnight with one of the following primary antibodies: anti-Iba-1 (1:1,000, Wako, Richmond, VA, USA), horseradish peroxidase-conjugated anti-mouse IgG (1:100; Pierce, Rockford, IL, USA), anti-cleaved caspase-3 (1:100; Cell Signaling, Danvers, MA, USA), anti-p-JNK (1:100; Cell Signaling, Danvers, MA, USA), anti-TNFR1 (1:200; Abcam, Cambridge, MA, USA), and anti-TNFR2 (1:100; Abnova, Walnut, CA, USA). After incubation with biotinylated secondary antibodies (anti-rabbit IgG 1:200; Pierce, Rockford, IL, USA), biotin-peroxidase signals were detected using 0.5 mg/mL 3′3′-diaminobenzidine (DAB)/0.003% H_2_O_2_ as a substrate. The results were recorded using a microscope (BX51; Olympus, Tokyo, Japan).

### Assessment of cortical and white matter injury

On P17 (12 days post-insult), the brains were post-fixed, dehydrated and embedded in paraffin, and then coronally sectioned (10-μm thick) from the genu of the corpus callosum to the end of the dorsal hippocampus. Three sections per brain as described above were assessed.

#### Cortical damage

Nissl-stained sections were scanned and the cortical areas were measured using ImagePro Plus 6.0 (Media Cybernetics, Bethesda, MD, USA). The percentage of area loss in the cortex of the ipsilateral versus the contralateral hemisphere was calculated [8].

#### White matter injury

White matter injury was evaluated by myelin basic protein (MBP) staining for myelination and glial fibrillary acidic protein (GFAP) staining for astrogliosis. After permealization and blocking of non-specific binding, sections were first incubated at 4°C overnight with the primary rat monoclonal anti-MBP antibody (1:100; Millipore, Billerica, MA, USA) or rabbit polyclonal anti-GFAP antibody (1:800; Millipore, Billerica, MA, USA), rinsed, and then incubated with biotinylated goat anti-rat (1:200; Santa Cruz Biotechnology, Santa Cruz, CA, USA) or anti-rabbit (1:300; Pierce Biotechnology, Rockford, IL, USA) IgG. Positively-stained cells were visualized using avidin-biotin-peroxidase complex amplification (Pierce Biotechnology, Rockford, IL, USA) with diaminobenzidine tetrahydrochloride detection. The MBP expression was assessed in 3 regions within the white matter in each hemisphere of each section at 100× magnification per visual field (0.579 mm^2^), and graded using a 4-point scoring system: 0, loss of processes and complete loss of the capsule; 1, loss of processes with thinning or breaks in the capsule; 2, complete loss of processes with intact capsule; 3, partial loss of processes; and 4, no MBP loss [8,9]. The scores of each region were summed up to obtain a total score (range, 0 to 12) for each hemisphere.

### Quantitative analysis of immunohistochemical staining

Measurements of the numbers of Iba-1 and cleaved caspase-3 positive cells, and of the integrated optical density (IOD) of IgG, GFAP, p-JNK, TNFR1 and TNFR2 signals, were performed at 200× magnification per visual field (0.145 mm^2^) and analyzed using ImagePro Plus 6.0 [8,9]. Three visual fields in the medial, middle, and lateral areas of the ipsilateral cortex per section, and three sections per brain as described above were analyzed. Iba1-positive cells with amoeboid morphology were counted as activated microglia for analysis. The mean IOD values in the ipsilateral hemisphere of each experimental group were compared to those of the control group to obtain the relative IOD ratios.

### Immunofluorescence

Mouse pups in the LPS + HI group were perfused at 24 hours post-insult. After blocking, the sections were incubated overnight at 4-°C with a mixture of 2 of the following primary antibodies: anti-mouse NeuN (1:100; Millipore, Billerica, MA, USA,), mouse monoclonal anti-O4 immunoglobulin M (IgM) (1:100; Millipore, Billerica, MA, USA), anti-isolectin IB4 Alexa Fluor 594 (1:200; Invitrogen, Carlsbad, CA, USA), anti-Iba-1 (1:1,000; Wako, Richmond, VA, USA), anti-cleaved caspase-3 (1:100; Cell Signaling, Danvers, MA, USA), anti-TNF-α (1:100; Biolegend, San Diego, CA, USA), anti-p-JNK (1:100; Cell Signaling, Danvers, MA, USA), and anti-TNFR1 (1:100; Santa Cruz Biotechnology, Santa Cruz, CA, USA). The sections were washed and then incubated with Alexa Fluor 594 or 488 secondary antibodies (1:400; Invitrogen, Carlsbad, CA, USA). The slides were photographed for red (Alexa Fluor 594) and green (Alexa Fluor 488) fluorescence with a fluorescent microscope (E400; Nikon Instech, Kawasaki, Japan).

### Statistical analysis

Statistical significance (*P* < 0.05) was determined using the Kruskal-Wallis test, and Dunn’s method was used for *post-hoc* comparisons. Continuous data were presented as means ± standard errors of mean (SEM).

## Results

### LPS sensitized hypoxic-ischemic injury in the immature brain

**Figure 1:**
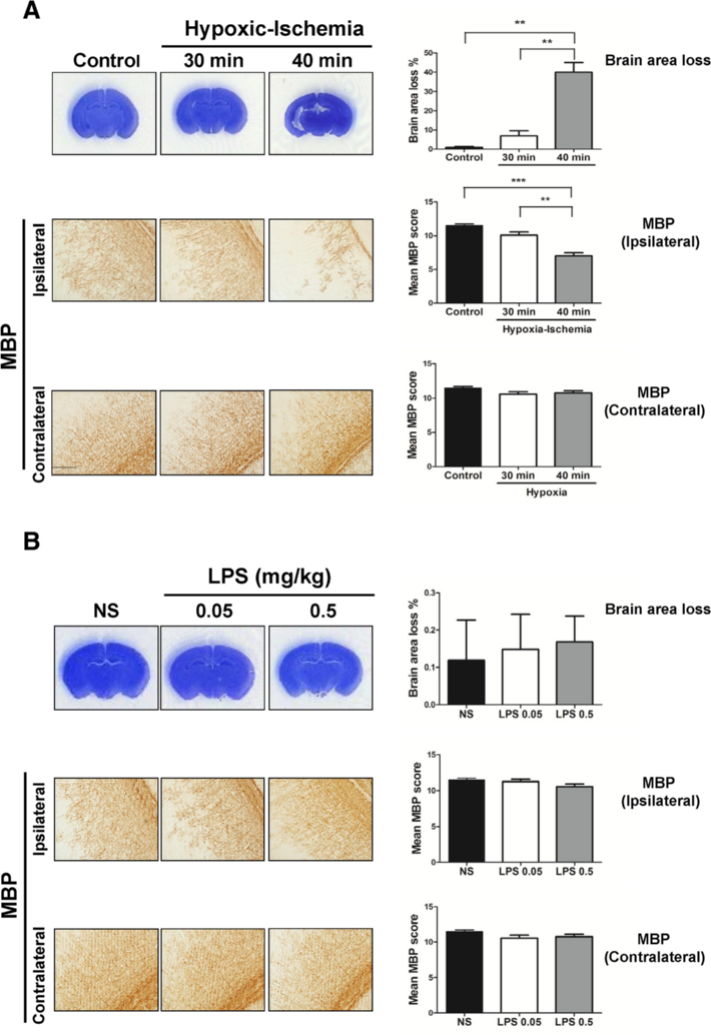
**Effects of hypoxic-ischemia (HI) or lipopolysaccharide (LPS) alone on brain injury.** Mouse pups were exposed to different duration of HI or different doses of LPS on P5, and neuropathological examinations were performed on P17. **(A)** Compared to the control groups (n = 6), pups exposed to 40 minute-HI (n = 9), but not to 30-minute HI (n = 10), had significant injury in the cortex (upper panel) and decreased myelin basic protein (MBP) expression in the white matter of the ipsilateral hemispheres (middle panel). There were no significant differences in MBP expression in the contralateral white matter among the control, 30-minute HI and 40-minute HI groups (lower panel). **(B)** Compared to the normal saline (NS) group (n = 5), the 0.05-mg/kg LPS (n = 5) and 0.5-mg/kg LPS (n = 5) groups showed no evident cortical (Nissl staining, upper panel) or white matter injury (MBP staining, middle and lower panels). Scale bar = 200 μm. Values are means ± SEM. **P < 0.01, ***P < 0.001.

The cortical and white matter injuries in P5 mouse pups exposed to different HI durations (30 and 40 minutes) were first determined. Neuropathologic examinations performed on P17 showed that the pups exposed to 40-minute (40% mortality) HI, but not to 30-minute (5.6% mortality) HI, had significant brain area loss (Figure 1A, upper panel) and reduced MBP expression (Figure 1A, middle panel) in the ipsilateral hemisphere compared to the control pups without HI exposure. The 30- and 40-minute HI and the control groups had similar MBP expression in the contralateral hemispheres (Figure 1A, lower panel). Thus, 30-minute HI was used for the LPS-sensitized HI experiments.

Next, we determined whether LPS injection alone induced brain injury. Neuropathologic examinations on P17 showed that the 0.05-mg/kg and 0.5-mg/kg LPS groups had no significant injury in the cortex (Figure 1B, upper panel) and white matter (Figure 1B, middle and lower panels) compared to the NS group. Then, we examined the LPS effect on HI mortality. After HI, the 0.5-mg/kg LPS group had significantly higher mortality than the 0.05-mg/kg LPS group (32.3% versus 13.1%, *P* < 0.05).

**Figure 2:**
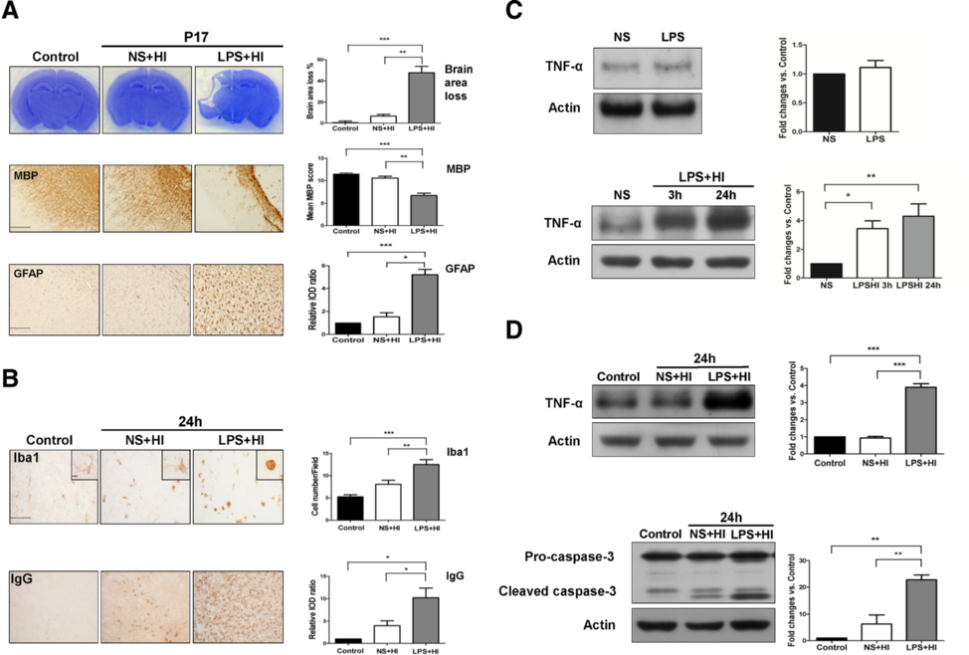
**Lipopolysaccharide (LPS)-sensitized hypoxic-ischemic (HI) injury induced up-regulation of neuroinflammation, blood–brain barrier damage and cell apoptosis. (A)** P5 mouse pups received normal saline (NS) or LPS (0.05 mg/kg) injection before 30-minute HI. Neuropathology was performed on P17. Compared to the control group (n = 6), the LPS + HI group (n = 10) but not the NS + HI group (n = 10) had significantly increased cortical damage (Nissl staining, upper panel), markedly reduced myelination (MBP, middle panel), and increased astrogliosis in the ipsilateral hemisphere (GFAP, lower panel). **(B)** At 24 hours post-insult, immunohistochemistry revealed that the LPS + HI group (n = 10) had significant increases in Iba1-positive microglia and IgG extravasation than the NS + HI (n = 10) and control (n = 6) groups. **(C)** LPS injection before HI did not up-regulate TNF-α expression compared to NS injection (upper panel). TNF-α levels were significantly increased at 3 hours, and especially at 24 hours after LPS-sensitized HI (lower panel). n = 4 experiments. **(D)** The LPS + HI group had significantly higher levels of TNF-α (upper panel) and cleaved caspase-3 (lower panel) than the NS + HI group at 24 hours post-insult. n = 6 experiments. Scale bar = 200 μm for MBP and = 100 μm for others. Inset scale bar = 10 μm in **(B)**. Values are means ± SEM. *P < 0.05, **P < 0.01, ***P < 0.001. GFAP, glial fibrillary acidic protein; Iba-1, ionized calcium-binding adaptor molecule-1; MBP, myelin basic protein.

Therefore, 0.05-mg/kg LPS was chosen for the following LPS-sensitized HI experiments on brain injury. On P17, the LPS + HI group had significantly more cortical damage, white matter injury with decreased MBP expression, and increased astrogliosis than the control and NS + HI groups (Figure 2A).

### LPS-sensitized hypoxic-ischemia up-regulated neuroinflammation and TNF-α, and worsened neurovascular damage

**Figure 3:**
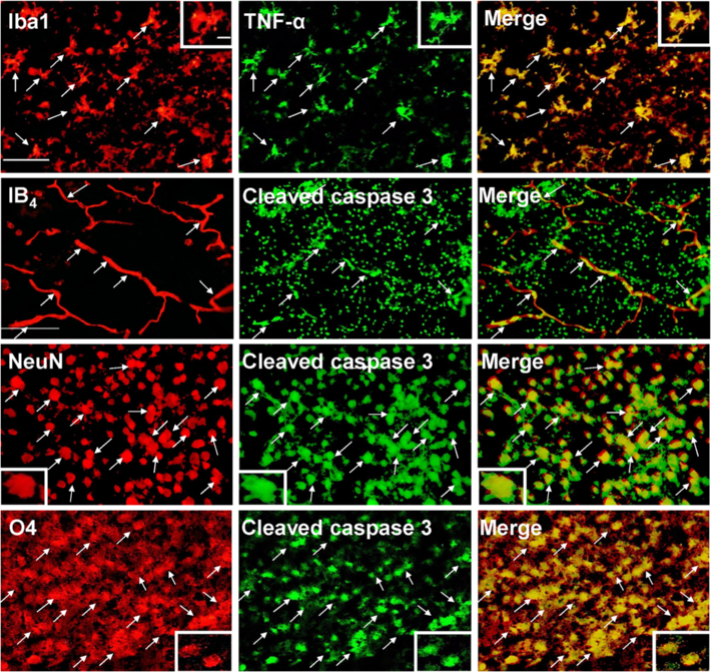
**TNF-α up-regulation, and apoptosis of endothelial cells, neurons, and oligodendrocyte progenitors after lipopolysaccharide (LPS)-sensitized hypoxic-ischemia (HI).** Immunofluorescence in the LPS + HI group 24 hours post-insult showed that many Iba1-positive activated microglia co-expressed TNF-α, whereas IB4-positive endothelial cells, NeuN-positive neurons, and O4-postive oligodendrocytes precursor cells co-expressed cleaved caspase-3. Scale bar = 50 μm for IB4 and 25 μm for others. Inset scale bar = 5 μm.

At 24 hours post-insult, the LPS + HI group, rather than the NS + HI group, had significantly increased Iba1-positive activated microglia and BBB damage (IgG extravasation) (Figure 2B). The effects of LPS and LPS + HI on TNF-α expression were examined using immunoblotting. Compared to NS, LPS injection before HI did not increase TNF-α levels (Figure 2C, upper panel). In contrast, after LPS-sensitized HI, the TNF-α levels were significantly increased at 3 hours, and especially at 24 hours after HI (Figure 2C, lower panel). The LPS + HI pups also had significantly more up-regulation of TNF-α (Figure 2D, upper panel) and cleaved caspase-3 levels (Figure 2D, lower panel) at 24 hours post-insult compared to the NS + HI pups. Further immunofluorescence study in the LPS + HI group showed that Iba1-positive microglia highly expressed TNF-α (Figure 3). Cleaved caspase-3 was highly expressed in the IB_4_-positive microvascular endothelial cells, NeuN-positive neurons, and O4-positive oligodendroglial precursor cells, indicating that these cells were undergoing apoptosis (Figure 3).

### LPS-sensitized hypoxic-ischemia induced JNK activation in microglia, microvascular endothelial cells, neurons, and oligodendrocyte progenitors

**Figure 4:**
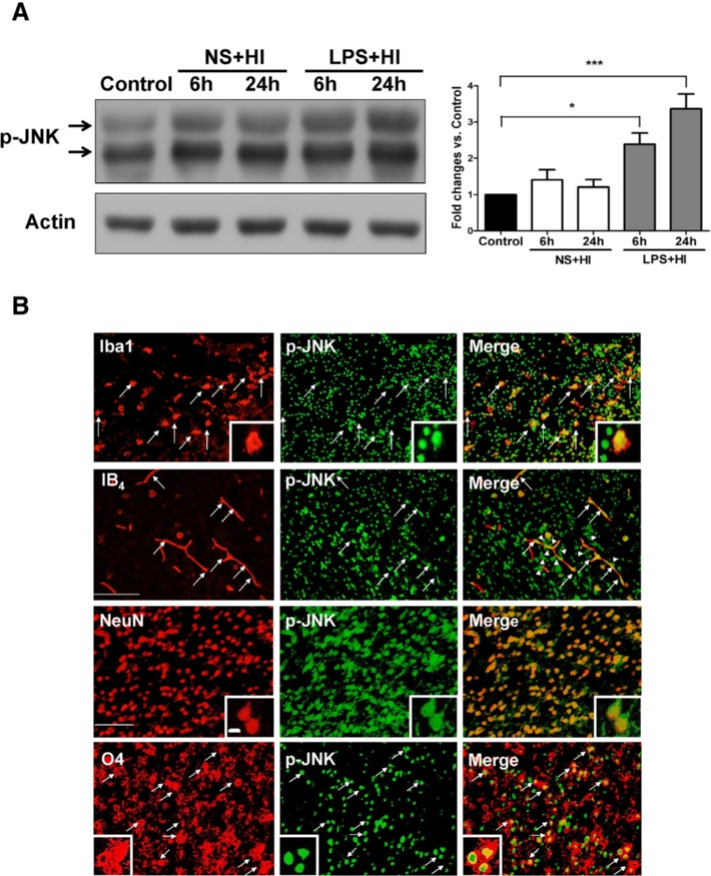
**Up-regulation of c-Jun N-terminal kinase (JNK) activation in microglia, endothelial cells, neurons, and oligodendrocyte progenitors after lipopolysaccharide (LPS)-sensitized hypoxic-ischemia (HI). (A)** Immunoblotting showed that the LPS + HI group (n = 4), but not the NS + HI group (n = 4) had increased p-JNK expression at 6 and 24 hours post-insult compared to the control group (n = 3). **(B)** Immunofluorescence in the LPS + HI group 24 hours post-insult showed up-regulation of p-JNK expression in Iba1-positive microglia, IB_4_-positive microvascular endothelial cells, NeuN-positive neurons, and O4-positive oligodendrocyte progenitors. Arrowheads indicate many p-JNK-positive cells attached to or were located around the IB_4_-positive microvessels. Scale bar = 50 μm for Iba1 and IB4, and 25 μm for others. Inset scale bar = 2.5 μm. Values are means ± SEM. *P < 0.05, ***P < 0.001.

Immunoblotting demonstrated persistent JNK activation at 6 and 24 hours post-insult in the LPS + HI group, but not in the NS + HI group (Figure 4A). Immunofluorescence study in the LPS + HI group further revealed up-regulation of p-JNK expression in activated microglia, microvascular endothelial cells, neurons, and oligodendrocyte progenitors at 24 hours post-insult (Figure 4B). In addition, there were many p-JNK-positive cells (green light) attached to or located around the IB_4_-positive microvessels (arrowheads in Figure 4B).

### TNF-α inhibition protected against LPS-sensitized hypoxic-ischemic brain injury

**Figure 5:**
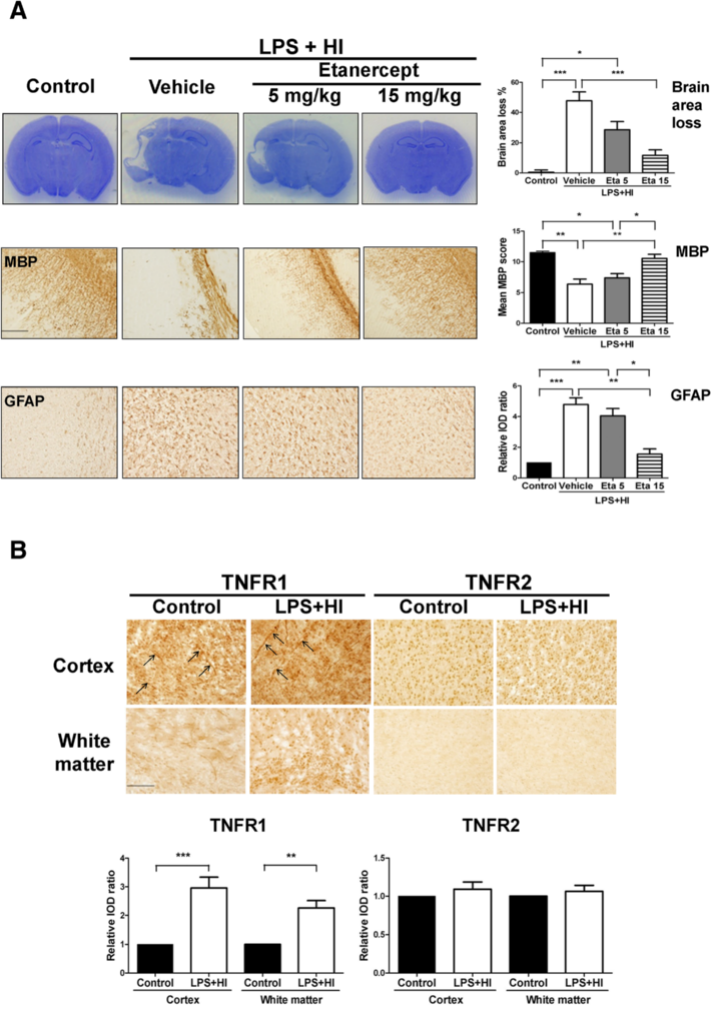
**TNF-α inhibition protected against lipopolysaccharide (LPS)-sensitized hypoxic-ischemic (HI) brain injury. (A)** Etanercept at a dosage of 15 mg/kg (n = 16), but not 5 mg/kg (n = 12), significantly attenuated cortical injury (Nissl staining, upper panel), preserved myelination (MBP, middle panel), and reduced astrogliosis (GFAP, lower panel) compared to the vehicle-treated group (n = 14) after LPS-sensitized HI on P17. **(B)** The LPS + HI groups (n = 8) had significantly increased immunoreactivity of TNFR1 but not TNFR2 in the cortex (upper panel) and white matter (lower panel) 24 hours post-insult compared to the control groups (n = 6). The up-regulated TNFR1 was expressed in blood vessels (arrows) and non-vascular cells. Scale bar = 200 μm for MBP and 100 μm for others. Values are means ± SEM. ***P < 0.001, **P < 0.01, *P < 0.05. GFAP, glial fibrillary acidic protein; MBP, myelin basic protein; TNFR, tumor necrosis factor receptor.

Compared to vehicle, etanercept significantly attenuated cortical injury at a dose of 15 mg/kg but not at 5 mg/kg treatment in the LPS + HI group, and preserved MBP expression and reduced astrogliosis in the white matter on P17 (Figure 5A).

### TNFR1 was up-regulated in microglia, microvascular endothelial cells, neurons, and oligodendrocyte progenitors after LPS-sensitized hypoxic-ischemia

**Figure 6:**
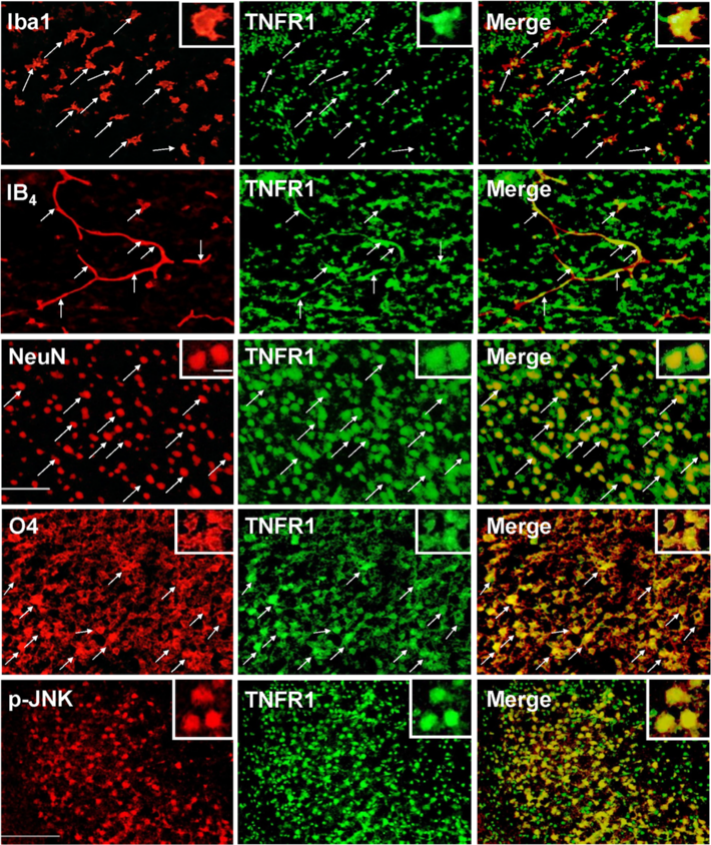
**Up-regulation of tumor necrosis factor receptor 1 (TNFR1) expression in microglia, endothelial cells, neurons, and oligodendrocyte progenitors after lipopolysaccharide (LPS)-sensitized hypoxic-ischemia (HI).** Immunofluorescence of the LPS + HI group 24 hours post-insult showed up-regulated TNFR1 expression in Iba1-positive microglia, IB_4_-positive microvascular endothelial cells, NeuN-positive neurons, and O4-positive oligodendrocyte progenitors. TNFR1 also co-localized with p-JNK. Scale bar = 25 μm for NeuN and O4, and 50 μm for others. Inset scale bar = 5 μm.

We further investigated which of the two TNF-α receptors (TNFR1 and TNFR2) played a predominant role in LPS-sensitized HI brain injury. Immunohistochemistry in the LPS + HI group showed significant up-regulation of TNFR1 but not of TNFR2 expression in the cortex and white matter 24 hours post-insult (Figure 5B). Immunofluorescence showed that TNFR1 was expressed mainly in the microglia, endothelial cells, neurons and oligodendrocyte progenitors, and most of the TNFR1-positive cells also co-expressed p-JNK (Figure 6).

### TNFR1 but not TNFR2 down-regulation reduced JNK activation, attenuated neuroinflammation and neurovascular damage, and ameliorated brain injury after LPS-sensitized hypoxic-ischemia

**Figure 7:**
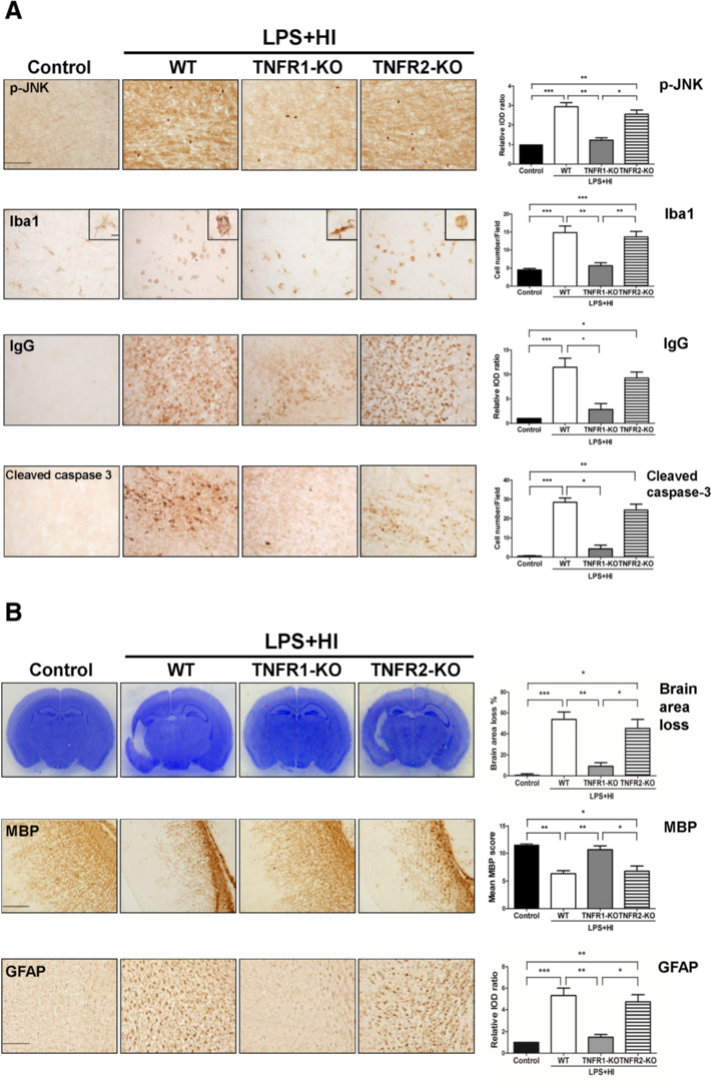
**Tumor necrosis factor receptor 1 (TNFR1) deficiency reduced c-Jun N-terminal kinase (JNK), microglial activation and blood–brain barrier (BBB) damage, and attenuated injury after lipopolysaccharide (LPS)-sensitized hypoxic-ischemia (HI). (A)** At 24 hours post-insult, the TNFR1- (n = 12) but not the TNFR2-KO (n = 12) pups had significant reduction in p-JNK expression and decreased Iba1-positive microglia, IgG extravasation, and cleaved caspase 3-positive cells than the WT pups (n = 10). **(B)** The TNFR1-KO but not the TNFR2-KO mice also had significantly reduced cortical injury (Nissl stain, upper panel), preserved myelination (MBP, middle panel), and decreased astrogliosis (GFAP, lower panel) than the WT mice on P17. GFAP, glial fibrillary acidic protein; Iba-1, ionized calcium-binding adaptor molecule-1; KO, knockout; MBP, myelin basic protein; WT, wild-type. Scale bar = 100 μm in (A), and 200 μm for MBP, 100 μm for GFAP in (B). Inset scale bar = 10 μm. Values are means ± SEM. ***P < 0.001, **P < 0.01, *P < 0.05.

The TNFR1-KO pups, but not the TNFR2-KO pups, had significantly reduced p-JNK expression, with decreased activated microglia, BBB breakdown, and cleaved caspase-3-positive cells compared to the wild-type pups at 24 hours post-insult (Figure 7A). On P17, the TNFR1-KO mice, but not the TNFR2-KO mice, had significant attenuation of cortical and white matter injury with decreased astrogliosis compared to the wild-type mice (Figure 7B).

### Suppression of JNK activation protected against LPS-sensitized hypoxic-ischemic brain injury

**Figure 8:**
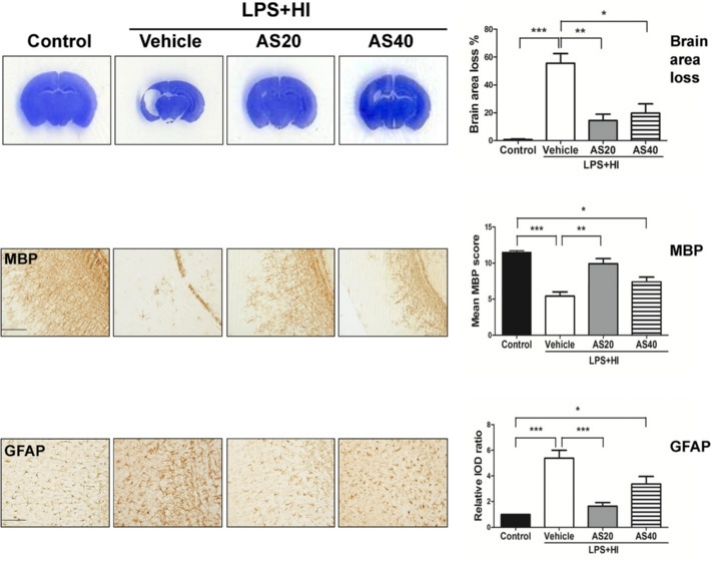
**Inhibiting c-Jun N-terminal kinase (JNK) activity using AS601245 significantly attenuated lipopolysaccharide (LPS)-sensitized hypoxic-ischemic (HI) brain injury.** AS601245 treatment at a dose of 20 mg/kg (n = 12) was more effective than 40 mg/kg (n = 11) in attenuating cortical injury (Nissl stain, upper panel), increasing myelination (MBP, middle panel), and decreasing astrogliosis (GFAP, lower panel) than vehicle treatment (n =15) on P17 after LPS-sensitized HI on P5. Scale bar = 200 μm for MBP and 100 μm for GFAP. Values are means ± SEM. ***P < 0.001, **P < 0.01, *P < 0.05. GFAP, glial fibrillary acidic protein; MBP, myelin basic protein.

The effect of JNK inhibition on LPS-sensitized HI injury was then examined using AS601245, an ATP-competitive inhibitor of JNK. Compared to vehicle, AS601245 treatment in the LPS + HI group at a dose of 20 mg/kg was more effective than a dose of 40 mg/kg in attenuating cortical damage, preserving MBP expression, and reducing astrogliosis on P17 (Figure 8).

## Discussion

Neonatal pre-exposure to systemic inflammation may affect cerebral vulnerability and thereby act concomitantly with HI insult to aggravate brain injury [31]. The present study demonstrated that P5 mouse pups, equivalent to very preterm infants in brain maturation status, subjected to the combination of LPS and HI had cortical and white matter injury on P17. The damage was characterized by apoptosis of neurons and oligodendrocyte progenitors, BBB disruption, and microglial activation in association with the selective up-regulation of TNFR1 and activation of JNK. Furthermore, the up-regulated TNFR1 and p-JNK were co-localized in the cellular component of the neurovascular unit (neurons, oligodendroglial precursors, and microvascular endothelial cells) and microglia. Inhibition of TNF-α or JNK activity exerted significant neuroprotection. More importantly, genetic deficiency of TNFR1, but not TNFR2, suppressed JNK activation, reduced neuronal and oligodendroglial apoptosis, attenuated BBB breakdown and microglial activation, and protected against cortical and white matter injury. These findings suggest that TNFR1-JNK signaling is a shared pathway linking neurovascular damage and neuroinflammation that contribute to LPS-sensitized HI injury in the immature brain.

Neurons and vascular cells are closely related developmentally and functionally [32]. Communication between the nervous and vascular systems is required to maintain the integrity of the BBB and promote neural function in the developing brain [33]. Neurons, oligodendrocyte progenitors, and microvascular endothelial cells form a close, inter-related neurovascular unit in the cortex and white matter, which may be the major targets of injury in the immature brain [8,9,11,12]. Damage to the neurons and microvasculature may occur progressively after HI [34]. After insults, damaged microvessels may recruit activated leukocytes into the injured brain through the disrupted BBB, leading to sustained neuroinflammation, which in turn further damages the brain and microvasculature through prolonged production of pro-inflammatory cytokines [15]. As such, neurovascular damage and neuroinflammation are two mutually-potentiating mechanisms leading to injury in the developing brain.

Previous studies have found that inflammation alone, even at moderate levels (repeated 0.01-mg/kg IL-β or 0.3-mg/kg LPS injection), caused injury in the developing brain [35,36]. The present study showed that a single LPS injection alone at a lower dose (0.05 mg/kg) did not induce brain injury. However, the low-dose LPS can sensitize neurovascular damage and worsen brain injury after HI via the TNFα-TNFR1-JNK pathway. Studies investigating the mechanisms of LPS sensitization demonstrate early up-regulation of genes that are associated with stress-induced inflammatory response and cell death in the neonatal brain several hours after peripheral exposure to LPS, with the priming effect contributing to increased vulnerability of the immature brain to subsequent insults [37,38]. The TNF-α pathway plays a key role in inflammation-sensitized excitotoxic brain injury in neonatal mice [27]. *TNF-α* gene cluster deletion abolishes LPS-mediated sensitization of the neonatal brain to HI insult [38]. Similarly, an *in vitro* study has shown that TNFR1 signaling is essential for LPS-induced sensitization to oxygen-glucose deprivation in murine neonatal organotypic hippocampal slices [39]. Another *in vitro* study also found that oxygen-glucose deprivation enhanced TNF-α/IFN-γ toxicity via up-regulation of TNF-related apoptosis-inducing ligand signaling in both neuronal and oligodendrocyte progenitor cell cultures [40].

Our previous study showed that JNK activation is involved in LPS-sensitized HI injury in the immature brain [9]. However, the upstream pathway leading to JNK activation remains unclear. Nijboer’s work showed JNK inhibition protected against HI brain injury in P7 rat pups [23]. They revealed that JNK inhibition did not reduce HI-induced cytokine expression including TNF-α, suggesting JNK might not only be one of the downstream pathways of TNF-α signaling but also involved in HI injury of the neonatal brain [23]. Our present study on the respective role of TNF-α, TNFR and JNK in LPS-sensitized HI injury of P5 mice demonstrates that TNF-α-triggered TNFR1-JNK activation is a critical shared pathway leading to neuroinflammation and neurovascular injury after LPS-sensitized HI in the immature brain.

Activated microglia play a central role as a converging point for upstream HI/inflammation and downstream cytotoxicity in the pathogenesis of neurovascular injury in the immature brain [1,14]. The present study demonstrates TNF-α, TNFR1, and JNK up-regulation, and microglial activation post-insult, with co-localization of TNFR1 and p-JNK in the activated microglia. A previous study has shown that JNK activation in microglia triggered by LPS-sensitized HI is associated with nuclear translocation of the downstream molecule c-Jun, suggesting the neuroinflammatory role of microglia [9]. p-JNK-positive activated microglia then release TNF-α, which may not only exert cytotoxic effects on endothelial cells, neurons, and oligodendrocyte progenitors, but also facilitate prolonged microglial activation by promoting JNK synthesis through TNFR1 in an autocrine loop [41].

Systemic application of LPS alone induces up-regulation of pro-inflammatory cytokines in P7 mouse forebrain in association with strong activation of microglia and microvessels [38]. Deletion of the entire *TNF* gene cluster not only greatly attenuates endotoxin-mediated increase in cerebral infarct volume after HI, but also prevents microglial and endothelial activation following application of LPS alone [38], suggesting the potential involvement of microglia and microvascular endothelial cells in LPS-mediated sensitization to neonatal brain injury.

The TNF-α and JNK signaling may contribute to leukocyte- and microglia-driven BBB disruption [42]. Animal experiments demonstrate that stroke and subarachnoid hemorrhage can up-regulate TNF-α or JNK signaling for BBB permeability [21,43]. *In vitro* studies also show the involvement of TNFR and JNK activation in the apoptosis of cerebral microvascular endothelial cells [44,45]. In this study, LPS-sensitized HI induced perivascular aggregation of p-JNK-positive cells, which may be endogenous brain cells or peripheral leukocytes infiltrating through the disrupted BBB. During detrimental insults, p-JNK-positive activated leukocytes migrating into the brain may not only cause sustained BBB disruption by enhancing TNFα-mediated matrix metalloproteinase-9 activity [46], but also result in prolonged activation of microglia, which in turn further damage the BBB through TNFR1 signaling by lasting TNF-α production and chemokine secretion to attract more leukocytes into the brain [15,47]. Therefore BBB breakdown may act in concert with activated microglia to worsen LPS-sensitized HI injury.

Autopsy studies of periventricular leukomalacia have shown enhanced TNF-α expression in cortical neurons and that pre-myelinating oligodendrocytes are the major apoptotic cells in the white matter [13,48]. TNFR1 is significantly up-regulated in apoptotic oligodendrocytes following hypoxia in neonatal rats [49]. *In vitro* studies also have shown that oligodendrocyte progenitors obtained from TNFR1/TNFR2-KO mice are resistant to LPS-induced microglial toxicity [50], and TNFR1 is necessary for apoptosis of oxygen-glucose deprived cortical neurons [51]. Moreover, JNK activation plays an important role in stress-induced apoptosis of oligodendrocyte progenitors and HI neuronal death [23,52]. In this neonatal mouse model, we showed that neurons, O4-positive oligodendrocyte precursor cells, and endothelial cells are the target of injury in the neurovascular unit after LPS-sensitized HI.

The co-localization of p-JNK and TNFR1 in apoptotic cells implicated the key role of TNFR1-JNK signaling in triggering death events in the neurovascular unit. The cytotoxic effects of TNF-α may be mediated directly through TNFR1 by formation of death-inducing signaling complex, or indirectly, via intrinsic/extrinsic apoptotic pathways induced by TNFR1-JNK activation [16,19]. In addition to cell death, surviving oligodendrocyte progenitors may be deterred from proliferation and differentiation by microglial activation and reactive astrocytes [1]. Our findings of reactive astrogliosis and hypomyelination on P17 after LPS-sensitized HI may imply the effects of neuroinflammation on impairment of oligodendroglial maturation.

## Conclusion

In summary, TNFR1-JNK signaling is up-regulated after LPS-sensitized HI and acts as a shared pathway leading to neurovascular damage and neuroinflammation, which may potentiate with each other to worsen cortical and white matter injury in the immature brain (Additional file 1: Figure S1). Blocking the loop of TNFR1-JNK signaling effectively protects against inflammation-sensitized HI injury in the immature brain. Thus, TNFR1-JNK signaling may emerge as a potential therapeutic target for brain injury in very preterm infants.

## Additional file

Additional file 1: Figure S1. A proposed diagram showing the self-potentiating loop of TNFR1-JNK signaling in the pathogenesis of inflammation-sensitized hypoxic-ischemic neurovascular injury in the immature brain. Lipopolysaccharide-sensitized hypoxic-ischemia may damage the neurovascular units (neurons, oligodendrocyte progenitors, microvascular endothelial cells) and activate microglia in the immature brain via a shared TNFR1-JNK signaling pathway leading to sustained neuroinflammation, blood–brain barrier disruption and cell apoptosis in a vicious cycle. The white arrows indicate possible roles of TNF-α in triggering microglial activation, blood–brain barrier breakdown and cell apoptosis in a self-augmenting loop. JNK, c-Jun N-terminal kinases; TNF-α, tumor necrosis factor-alpha; TNFR1, TNF-α receptor 1.

## Abbreviations

BBB: blood–brain barrier
DAB: 3′3′-diaminobenzidine
DMSO: dimethyl sulfoxide
GFAP: glial fibrillary acidic protein
HI: hypoxic-ischemia
Iba-1: ionized calcium-binding adaptor molecule-1
IFN: interferon
IgG: immunoglobulin G
IgM: immunoglobulin M
IOD: integrated optical density
ip: intraperitoneal
JNK: c-Jun N-terminal kinase
KO: knockout
LPS: lipopolysaccharide
MBP: myelin basic protein
NS: normal saline
P: postpartum
PBS: phosphate-buffered saline
p-JNK: phospho-JNK
TNF-α: tumor necrosis factor-α
TNFR1: tumor necrosis factor receptor 1
TNFR2: tumor necrosis factor receptor 2

## References

[1] Volpe JJ (2011). Systemic inflammation, oligodendroglial maturation and encephalopathy of prematurity. Ann Neurol.

[2] Leviton A, Paneth N, Reuss ML, Susser M, Allred EN, Dammann O, Kuban K, Marter LJ, Pagano M, Hegyi T, Hiatt M, Sanocka U, Shahrivar F, Abiri M, Disalvo D, Doubilet P, Kairam R, Kazam E, Kirpekar M, Rosenfeld D, Schonfeld S, Share J, Collins M, David Genest D, Debra Heller D, Schwarz SS (1999). Maternal infection, fetal inflammatory response, and brain damage in very low birth weight infants. Pediatr Res.

[3] Debillon T, Gras-Leguen C, Vérielle V, Winer N, Caillon J, Rozé JC, Gressens P (2000). Intrauterine infection induces programmed cell death in rabbit periventricular white matter. Pediatr Res.

[4] Yanowitz TD, Jordan JA, Gilmour CH, Towbin R, Bowen A, Roberts JM, Brozanski BS (2002). Hemodynamic disturbances in premature infants born after chorioamnionitis: association with cord blood cytokine concentrations. Pediatr Res.

[5] Wong FY, Silas R, Hew S, Samarasinghe T, Walker AM (2012). Cerebral oxygenation is highly sensitive to blood pressure variability in sick preterm infants. PLoS One.

[6] Kaukola T, Herva R, Perhomma M, Paakko E, Kingsmore S, Vainionpaa L, Hallman M (2006). Population cohort associating chorioamnionitis, cord inflammatory cytokines and neurological outcome in very preterm, extremely low birth weight infants. Pediatr Res.

[7] Wang LW, Lin YC, Wang ST, Yeh TF, Huang CC (2014). Hypoxic/ischemic and infectious events have cumulative effects on the risk of cerebral palsy in very-low-birth-weight preterm infants. Neonatology.

[8] Wang LW, Chang YC, Lin CY, Hong JS, Huang CC (2010). Low-dose lipopolysaccharide selectively sensitizes hypoxia-ischemia-induced white matter injury in the immature brain. Pediatr Res.

[9] Wang LW, Tu YF, Huang CC, Ho CJ (2012). JNK signaling is the shared pathway linking neuro-inflammation, blood–brain barrier disruption, and oligodendroglial apoptosis in the white matter injury of the immature brain. J Neuroinflammation.

[10] del Zoppo GJ (2006). Stroke and neurovascular protection. N Engl J Med.

[11] Tu YF, Tsai YS, Wang LW, Wu HC, Huang CC, Ho CJ (2011). Overweight worsens apoptosis, neuroinflammation and blood–brain barrier damage after hypoxic ischemia in neonatal brain through JNK hyperactivation. J Neuroinflammation.

[12] Tu YF, Lu PJ, Huang CC (2012). Moderate dietary restriction reduces p53-mediated neurovascular damage and microglia activation after hypoxic ischemia in neonatal brain. Stroke.

[13] Back SA, Luo NL, Borenstein NS, Levin JM, Volpe JJ, Kinney HC (2001). Late oligodendrocyte progenitors coincide with the developmental window of vulnerability for human perinatal white matter injury. J Neurosci.

[14] Chew LJ, Takanohashi A, Bell M (2006). Microglia and inflammation: impact on developmental brain injuries. Ment Retard Dev Disabil Res Rev.

[15] Dammann O, Durums S, Leviton A (2001). Do white cells matter in white matter damage?. Trends Neurosci.

[16] Manning AM, Davis RJ (2003). Target JNK for therapeutic benefit: from Junk to gold?. Nat Rev Drug Discov.

[17] Kadhim H, Tabarki B, Verellen G, De Prez C, Rona AM, Sebire G (2001). Inflammatory cytokines in the pathogenesis of periventricular leukomalacia. Neurology.

[18] Kadhim H, Khalifa M, Deltenre P, Casimir G, Sebire G (2006). Molecular mechanisms of cell death in periventricular leukomalacia. Neurology.

[19] Varfolomeev EE, Ashkenazi A (2004). Tumor necrosis factor: an apoptosis JuNKie?. Cell.

[20] Fontaine V, Mohand-Said S, Hanoteau N, Fuchs C, Pfizenmaier K, Eisel U (2002). Neurodegenerative and neuroprotective effects of tumor necrosis factor (TNF) in retinal ischemia: opposite roles of TNF receptor 1 and TNF receptor 2. J Neurosci.

[21] Pan W, Kastin A (2007). Tumor necrosis factor and stroke: role of the blood–brain barrier. Prog Neurobiol.

[22] Works MG, Koenig JB, Sapolsky RM (2013). Soluble TNF receptor 1-secreting ex vivo-derived dendritic cells reduce injury after stroke. J Cereb Blood Flow Metab.

[23] Nijboer CH, van der Kooij MA, van Bel F, Ohl F, Heijnen CJ, Kavelaars A (2010). Inhibition of the JNK/AP-1 pathway reduces neuronal death and improves behavioral outcome after neonatal hypoxic-ischemic brain injury. Brain Behav Immun.

[24] Rice JE, Vannucci RC, Brierley JB (1981). The influence of immaturity on hypoxic-ischemic brain damage in the rat. Ann Neurol.

[25] Zhu C, Wang X, Xu F, Bahr BA, Shibata M, Uchiyama Y, Hagberg H, Blomgren K (2005). The influence of age on apoptotic and other mechanisms of cell death after cerebral hypoxia-ischemia. Cell Death Differ.

[26] McCoy MK, Tansey MG (2008). TNF signaling inhibition in the CNS: implications for normal brain function and neurodegenerative disease. J Neuroinflammation.

[27] Aden U, Favrais G, Plaisant F, Winerdal M, Felderhoff-Mueser U, Lampa J, Lelievre V, Gressens P (2010). Systemic inflammation sensitizes the neonatal brain to excito-toxicity through a pro-/anti-inflammatory imbalance: key role of TNF-α pathway and protection by etanercept. Brain Behav Immun.

[28] Carboni S, Hiver A, Szyndralewiez C, Gaillard P, Gotteland JP, Vitte PA (2004). AS601245 (1,3-benzothiazol-2-yl (2-{[2-(3-pyridinyl) ethyl] amino}-4 pyrimidinyl) acetonitrile): a c-Jun NH2-terminal protein kinase inhibitor with neuro-protective properties. J Pharmacol Exp Ther.

[29] Paxinos G, Watson C (2001). The Mouse Brain in Stereotaxic Coordinates.

[30] Svedin P, Hagberg H, Savman K, Zhu C, Mallard C (2007). Matrix metalloproteinase-9 gene knock-out protects the immature brain after cerebral hypoxia-ischemia. J Neurosci.

[31] Hagberg H, Gressens P, Mallard C (2012). Inflammation during fetal and neonatal life: implications for neurologic and neuropsychiatric disease in children and adults. Ann Neurol.

[32] Tam SJ, Watts RJ (2010). Connecting vascular and nervous system development: angiogenesis and the blood–brain barrier. Annu Rev Neurosci.

[33] Quaegebeur A, Lange C, Carmeliet P (2011). The neurovascular link in health and disease: molecular mechanisms and therapeutic implications. Neuron.

[34] Hsu YC, Chang YC, Lin YC, Sze CI, Huang CC, Ho CJ (2014). Cerebral micro-vascular damage occurs early after hypoxia-ischemia via nNOS activation in neonatal brain. J Cereb Blood Flow Metab.

[35] Favrais G, van de Looij Y, Fleiss B, Ramanantsoa N, Bonnin P, Stoltenburg-Didinger G, Lacaud A, Saliba E, Dammann O, Gallego J, Sizonenko S, Hagberg H, Vincent L, Gressens P (2011). Systemic inflammation disrupts the developmental program of white matter. Ann Neurol.

[36] Rousset CI, Chalon S, Cantagrel S, Bodard S, Andres C, Gressens P, Saliba E (2006). Maternal exposure to LPS induces hypomyelination in the internal capsule and programmed cell death in the deep gray matter in newborn rats. Pediatr Res.

[37] Eklind S, Hagberg H, Wang X, Savman K, Leverin AL, Hedtjarn M, Mallard C (2006). Effect of lipopolysaccharide on global gene expression in the immature rat brain. Pediatr Res.

[38] Kendall GS, Hirstova M, Horn S, Dafou D, Acosta-Saltos A, Almolda B, Zbarsky V, Rumajogee P, Heuer H, Castellano B, Pfeffer K, Nedospasov SA, Peebles DM, Raivich G (2011). TNF gene cluster deletion abolishes lipopolysaccharide-mediated sensitization of the neonatal brain to hypoxic ischemic insult. Lab Invest.

[39] Markus T, Cronberg T, Cilio C, Pronk C, Wieloch T, Ley D (2009). Tumor necrosis factor receptor-1 is essential for LPS-induced sensitization and tolerance to oxygen-glucose deprivation in murine neonatal organo-typic hippocampal slices. J Cereb Blood Flow Metab.

[40] Kichev A, Rousset C, Baburamani AA, Levison SW, Wood TL, Gressens P, Thornton C, Hagberg H (2014). Tumor necrosis factor-related apoptosis-inducing ligand (TRAIL) signaling and cell death in the immature central nervous system after hypoxia-ischemia and inflammation. J Biol Chem.

[41] Kuno R, Wang J, Kawanokuchi J, Takeuchi H, Mizuno T, Suzumura A (2005). Autocrine activation of microglia by tumor necrosis factor-α. J Neuroimmunol.

[42] Rosenberg GA (2002). Matrix metalloproteinases in neuroinflammation. Glia.

[43] Yatsusshige H, Ostrowski RP, Tsubokawa T, Colohan A, Zhang JH (2007). Role of c-Jun N-terminal Kinase in early brain injury after subarachnoid hemorrhage. J Neurosci Res.

[44] Lucas R, Garcia I, Donati YRA, Hribar M, Mandriota SJ, Giroud C, Buurman WA, Fransen L, Suter PM, Nunez G, Pepper MS, Grau GE (1998). Both TNF receptors are required for direct TNF-mediated cytotoxicity in microvascular endothelial cells. Eur J Immunol.

[45] Karashi H, Michelsen KS, Arditi M (2009). Lipopolysaccharide-induced apoptosis in transformed bovine brain endothelial cells and human dermal microvessel endothelial cells: the role of JNK. J Immunol.

[46] Hosomi N, Ban CR, Naya T, Takahashi T, Guo P, Song XY, Kohno M (2005). Tumor necrosis factor-α neutralization reduced cerebral edema through inhibition of matrix metalloproteinase production after transient focal cerebral ischemia. J Cereb Blood Flow Metab.

[47] D’Mello C, Le T, Swain MG (2009). Cerebral microglia recruit monocytes into the brain in response to tumor necrosis factor-α signaling during peripheral organ inflammation. J Neurosci.

[48] Kadhim H, Tabarki B, De Prez C, Sebire G (2003). Cytokine immunoreactivity in cortical and subcortical neurons in periventricular leukomalacia: are cytokines implicated in neuronal dysfunction in cerebral palsy?. Acta Neuropathol.

[49] Deng YY, Lu J, Sivakumar V, Ling EA, Kaur C (2008). Amoeboid microglia in the peri-ventricular white matter induce oligodendrocyte damage through expression of pro-inflammatory cytokines via MAP kinase signaling pathway in hypoxic neonatal rats. Brain Pathol.

[50] Li J, Ramenaden ER, Peng J, Koito H, Volpe JJ, Rosenberg PA (2008). Tumor necrosis factor-α mediates lipopolysaccharide-induced microglial toxicity to developing oligodendrocytes when astrocytes are present. J Neurosci.

[51] Badiola N, Malagelada C, Llecha N, Hidalgo J, Comella JX, Sabria J, Rodriguez-Alvarez J (2009). Activation of caspase-8 by tumor necrosis factor receptor 1 is necessary for caspase-3 activation and apoptosis in oxygen-glucose deprived cultured cortical cells. Neurobiol Dis.

[52] Pirianov G, Jesurasa A, Mehmet H (2006). Developmentally regulated changes in c-Jun N-terminal kinase signaling determine the apoptotic response of oligodendrocyte lineage cells. Cell Death Differ.

