# Supplementary figures and images for: TNFR1-JNK signaling is the shared pathway of neuroinflammation and neurovascular damage after LPS-sensitized hypoxic-ischemic injury in the immature brain
Source: J Neuroinflammation. 2014 Dec 24;11:215. doi: 10.1186/s12974-014-0215-2 (PMC4300587; doi:10.1186/s12974-014-0215-2)

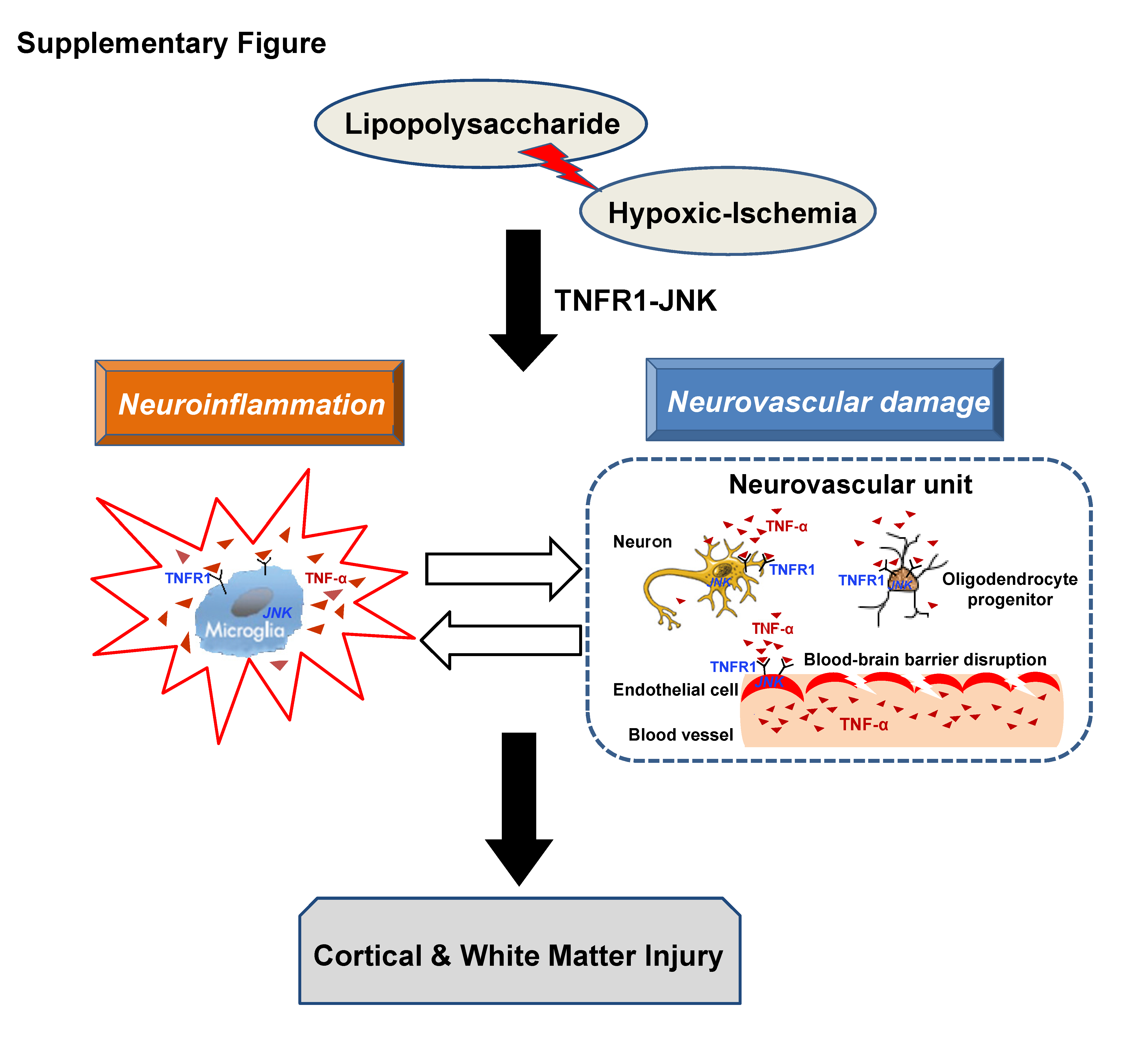

Supplement: Additional file 1: Figure S1. — A proposed diagram showing the self-potentiating loop of TNFR1-JNK signaling in the pathogenesis of inflammation-sensitized hypoxic-ischemic neurovascular injury in the immature brain. Lipopolysaccharide-sensitized hypoxic-ischemia may damage the neurovascular units (neurons, oligodendrocyte progenitors, microvascular endothelial cells) and activate microglia in the immature brain via a shared TNFR1-JNK signaling pathway leading to sustained neuroinflammation, blood–brain barrier disruption and cell apoptosis in a vicious cycle. The white arrows indicate possible roles of TNF-α in triggering microglial activation, blood–brain barrier breakdown and cell apoptosis in a self-augmenting loop. JNK, c-Jun N-terminal kinases; TNF-α, tumor necrosis factor-alpha; TNFR1, TNF-α receptor 1. [file 12974_2014_215_MOESM1_ESM.tiff]
